# Annotations

**Published:** 1900-12-15

**Authors:** 


### ANNOTATIONS.

### Arsenic in Medicine.

The disastrous consequences which have followed the widespread impregnation of beer with small quantities of arsenic should make some of us who are in the daily habit of ordering arsenic for our patients pause and think for a moment what may be the results of the prolonged use of such poison-containing prescriptions. A case has lately been recorded in which a man who had been a teetotaller for many months took again to drinking beer, and after consuming 36 pints in the course of three days quickly developed the now well-recognised symptoms of arsenical neuritis. It would thus appear that these symptoms were produced by a dose which probably did not exceed half a grain of arsenious acid. Suppose this to be so, let us consider what sort of doses of this drug are commonly employed therapeutically. The *British Pharmacopœia* gives a dose of Fowler's solution as being from 2 to 8 minims, and states that 110 minims contain 1 grain of arsenious anhydride. Taking the dose, then, at the maximum of 8 minims, and giving it three times a day, as is commonly enough done, we arrive at this position, that a patient would in a little over two days take as much arsenic as is presumed to have set up symptoms of arsenical poisoning in the unfortunate teetotaller just referred to. Of course 8 minims is a somewhat outside dose. But take the very ordinary dose of 5 minims three times a day, or take a very commonly sold preparation of Blaud's pill which contains a thirtieth of a grain of arsenious acid in each dose, and it is evident that many and many a dyspeptic or anæmic patient is taking day after day for really long periods an amount of arsenic quite enough, according to the testimony of the present epidemic, to set up severe symptoms, or if not to cause symptoms, at least to have an effect which may be far from salutary. Arsenic is very largely used in the treatment of skin diseases, of nerve diseases, of various forms of dyspepsia, and as a preventative of the skin manifestation of bromism; and although the extent to which it has been so used without obvious ill effects is a sufficient guarantee that in the majority of cases its administration for these purposes is harmless, it must be remembered that it is not every drinker of arsenical beer who has suffered from arsenical poisoning. There is an idiosyncrasy in these matters, and just as only here and there, out of the enormous crowd of beer drinkers, does one become affected with neuritis, so only here and there probably does a patient suffer from his medicine. Considering the doses given, however, and the readiness so often shown by patients to continue almost indefinitely the use of any prescription which has appeared to relieve the particular symptom for which it was prescribed, it would be very strange if, taken altogether, there were not, scattered over the country at the present time, a considerable number of people suffering in various ways not from "arsenic in beer," but from "arsenic in medicine."

### Tuberculosis in Prisons.

It is curious and it is interesting to notice how far-reaching are the effects of the general acceptance which has been given to modern views as to the communicability of tuberculosis. Every possible mode of life is being considered in reference to its influence in predisposing to this disease, and among others the life of the prisoner has now come under discussion. It is being said, and no one can deny its justice, that while society has a perfect right to inflict upon an evildoer such terms and forms of punishment as the sentence passed upon him demands, we have no right to make these punishments a lottery, a mere lucky-bag out of which one man may draw a penalty of a few months of somewhat uncomfortable board and lodging, while another may draw a sentence of death from tuberculosis.

Tuberculosis will probably always be more common among criminals than among the rest of the population. Not only does consumption lead to poverty, which too often drives to crime, but this disease may almost be regarded as the natural termination of that life of mingled vice and hardship which is led by those who—often from lack of an initial bodily or mental stamina—take to a career of crime. All the more necessary then that every care should be taken to prevent prisons becoming what it is their natural tendency to become, namely, hotbeds of tuberculosis; and in dealing with such a tainted population as that from which the inhabitants of our gaols are drawn, it is clear that the careful separation of prisoner from prisoner, as is done in so many of our English prisons, is one of the first things to be attended to. To the many things which can be said in favour of that form of punishment which has been so much railed against under the title of solitary confinement, is now to be added the fact that it does at least save a prisoner from many of those chances of infection with tuberculosis which can hardly be avoided in associated gaols. Attention has lately been drawn in America to the enormous mortality from tuberculosis which prevails in some of the penitentiaries in the States, and there seems much reason to believe that this arises in large part from the system of association which is there so common. It has been pointed out again and again that associated imprisonment is not only utterly unfair, in that what to some is hardly a punishment is to others an unspeakable degradation, but that its tendency is to drag all down to the same level and render reclamation next to impossible. Now we have added to these drawbacks the fact that unless associated imprisonment be coupled with a rigidity of discipline which must be very difficult to enforce, it exposes convicts to a danger from tuberculosis which certainly was never contemplated in their sentences.

### The Communion Cup.

The frequency with which the question is raised as to the possibility of disease being transmitted from person to person through the intermediary of the cup used in the administration of the holy communion renders it evident that the subject is not being considered as a mere medical curiosity, or as an illustration of the strange by-ways followed by infection, but that it is being regarded with serious anxiety among religious people, partly in reference to the dangers which they themselves may run from this source of infection, but much more largely, as we believe, in consequence of the fear lest in a fastidious generation any suspicion as to the cleanliness of a rite which is held to be one of the highest of religious privileges should hold waverers back from accepting a creed of which such things form part. The question then is one of much wider import, and is one which is being considered from a much more serious standpoint than the more light-hearted amongst us might imagine. As to the fact of infection having been transmitted in this way we fancy that the proofs, if there are any, must be few and far between; but as to the possibility of such a thing no medical man who is familiar with what is happening all round can have a moment's doubt; and it must be apparent to many that the cult of the unwashed and the efforts to make the lowest of the low full participators in all that the ritual of the Church offers for their comfort and for the sustenance of their faith, which have been so marked a feature of church life during the past score of years or more, have had the effect of imposing upon the "nicer" members of many congregations a close relationship with many most undesirable co-religionists, which certainly can only be described as "nasty." We will not enter into the various devices which have been proposed from time to time with the object of conforming at the same time with the words of the Church Service and with the dictates of cleanliness, some of which it would not be easy to discuss without undue levity. It seems to us, however, that just as the "bread" is placed upon the palm and swallowed without further handling, so there ought to be no difficulty in conveying a drop of the liquid to the recipient without the lips coming in contact with the vessel. The trouble arises chiefly in regard to the true meaning and conception of the verb to drink, and it ought not to be impossible to get over this by some means which would follow the words of the service and yet, from a sanitary point of view, would be without offence.

### Scattered Homes for Poor Law Children.

Regarding as we do with interest and with sympathy every movement which tends to make the pauper child like other children and to remove from him every trace of the stigma which attaches to the workhouse child, we cannot but agree with Earl Grey's criticisms of the conditions imposed by the Local Government Board in regard to the erection of scattered homes for Poor Law children. He urges that such children in normal health should not be better housed than the families of ordinary respectable working men, and that a so-called "scattered home" containing, as it should do, not more than about eight or ten children, under the care of a woman who would act as "mother" to them, ought to be like a workman's house and not like a middle-class villa. Instead of this, however, we find that the Local Government Board requires in new buildings erected for this purpose, a greater area than for ordinary cottages, special arrangements for ventilation, and a south aspect for sleeping and living rooms, and demands a fairly large garden, and that the stairs of such a house must be constructed of teak. Now we admit that all these are good things, and we would that all of us could be so favoured; but seeing as we do see every day of our lives the kind of houses and the sort of surroundings in which the children of respectable working people are brought up, conditions sanctioned and permitted by sanitary authorities which work under this self-same Local Government Board, we cannot admit the justice or the policy of enforcing a higher standard for Poor Law children than the standard which is passed and permitted in the case of others. So far as justice is concerned, either the standard demanded for the children of the State is far too high, or that permitted in regard to the children of the poor is too low, probably the latter; but so far as policy goes it is obvious enough that the standard should be the same, and that if the object of removing the children from great establishments and bringing them up in scattered homes is to take away the "workhouse taint," to make them like other children, and to restore them to their places in society, the "scattered homes" should be as like as possible to the homes of the people into whose ranks they are to be absorbed.

### Something Like a Jockey Club!

There is a thoroughness and completeness about some American institutions which is worthy of the imitation as well as of the envy of the inhabitants of these benighted isles. If report is to be credited—and in such matters we must confine ourselves to hearsay—we owe to the United States many new and interesting developments in the truly British sport of horse-racing. Not only in the attitude of the rider, but in the methods of inciting the horse to put on steam, English practice is said to be quite out of it, so that it is hardly surprising to hear that in another direction, namely, in the equipment of the racecourse, we are also being outdone. In a letter which appears in the *Medical Record* of New York we find a description of the hospital which is provided by the Pacific Jockey Club at the San Francisco racecourse, or, as it is called there, "race-track." This is not a mere ambulance station, at which "first aid" may be given to any jockey who may happen to be hurt, but a properly furnished little hospital containing "a clean modern operating room, with sterilisers, instruments, operating table, and such apparatus as one would expect to have in a well-conducted cottage hospital; a ward with beds completely equipped; a dispensary well stocked with drugs; and a storeroom containing splints, crutches, and surgical dressings." At this hospital, we are told, a regular surgeon is employed whose duty it is to be present during the racing, and give his undivided attention to anyone who may be injured or ill. American thoroughness, however, does not seem to stop at providing a hospital on the race-track, for we are told that this most useful and philanthropic Jockey Club no only looks after the physical welfare of its employés, but interests itself in their mental advancement by providing for their use a night school with a staff of instructors. This is something like a Jockey Club!

